# Placental Transmogrification of the Lung in a Patient Without Emphysematous Disease

**DOI:** 10.7759/cureus.53294

**Published:** 2024-01-31

**Authors:** Atl Simon Arias Rivera, Natalia De la Maza, Mauricio Damian Gomez Gonzalez, Jesus Vazquez, Alain Ledu Lara, Moises C Calderon Abbo

**Affiliations:** 1 General Surgery, Hospital Angeles Lomas, Huixquilucan, MEX; 2 Family Medicine, Hospital Angeles Lomas, Huixquilucan, MEX; 3 Cardiac Surgery, Hospital Angeles Lomas, Huixquilucan, MEX; 4 Pulmonology, Hospital Angeles Lomas, Huixquilucan, MEX; 5 Cardiothoracic Surgery, Hospital Angeles Lomas, Huixquilucan, MEX

**Keywords:** thoracic ct scan, pulmonary bulla, minimally invasive lung resection, thorax surgery, pulmonary placental transmogrification

## Abstract

We present a 47-year-old male without a relevant history or past respiratory diseases. He debuted with an acute, non-complicated COVID-19 infection, and later he started with mMRC-2 dyspnea, accompanied by a non-expectorant cough of four months evolution. A CT thoracic scan showed a dilatation of the aerial homogenous space and a well-defined anterior left pericardiac level, and a pericardial left bulla was diagnosed. The patient was treated with surgical intervention by video-assisted thoracoscopic surgery and had an adequate post-surgical evolution. The PPT must be managed by a multidisciplinary team with the definitive treatment of surgical resection.

## Introduction

Placental transmogrification of the lung was described for the first time in 1979. It is an extremely infrequent and benign lung disease, with only 40 cases reported in the literature, whose etiology is still unknown [1]. The word transmogrification means the act of changing into a different form or appearance, whether it is vegetable, animal, mineral, or human [2].

Due to the histological similarity to immature placental villi, the term placental could be the most appropriate to describe its characteristics, either histological or macroscopical. Microscopy shows characteristically papillary structures coated with hyperplastic pneumocytes, which resemble placental villi [3-4].

This disease normally presents as a giant, solitary bulla. Due to the rarity of the pathology, we present a case of a young male who consulted with dyspnea for four months and was treated with surgical management with a thoracoscopy. The postoperative diagnosis included this particular entity [1-2].

## Case presentation

**Figure 1:**
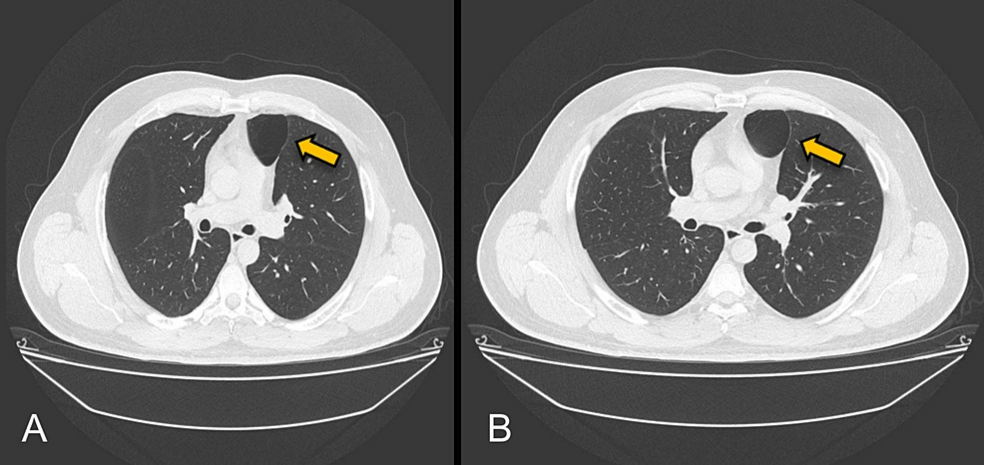
(A-B) Dilatation of the aerial homogenous space and a well-defined anterior left pericardiac level of 4.6 cm × 4.4 cm × 3.5 cm dimensions of thin wall, with a 1 mm thickness (yellow arrow)

**Figure 2:**
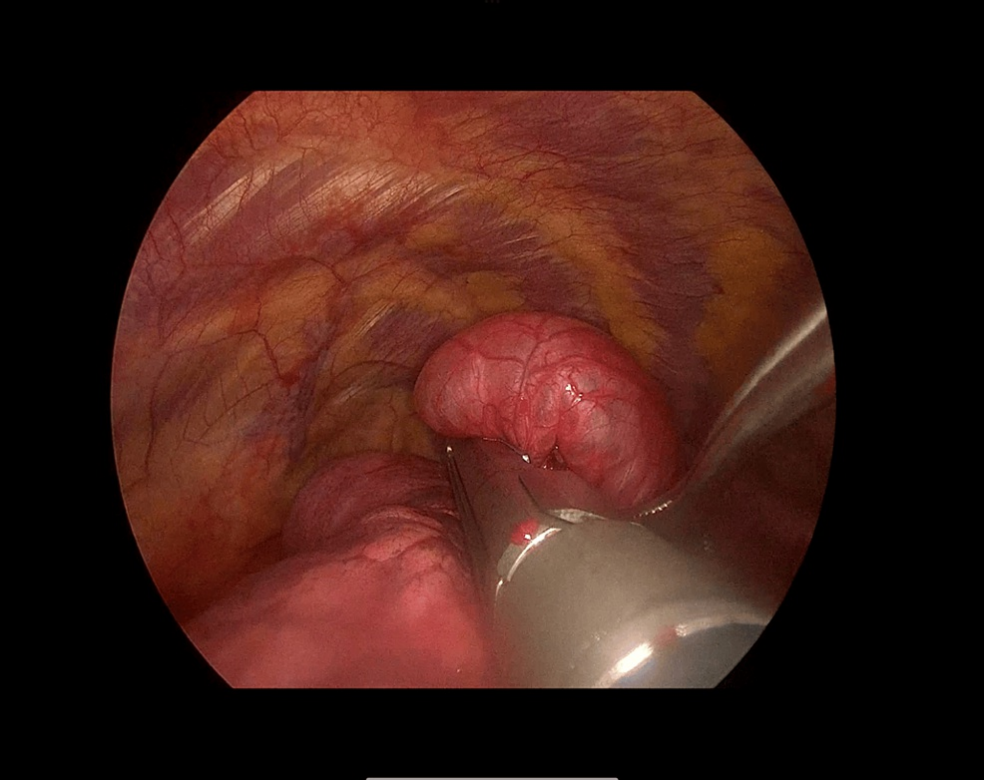
Placement of laparoscopic stapler in anterior segment of left lung

**Figure 3:**
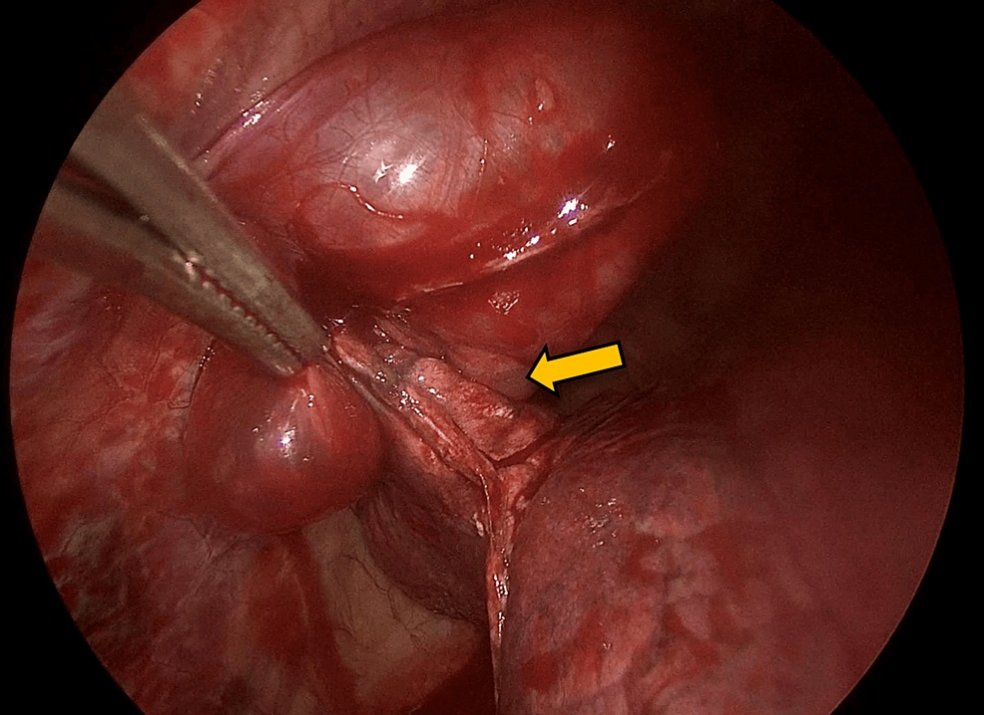
Laparoscopic stapler line posterior to wedge resection (yellow arrow)

**Figure 4:**
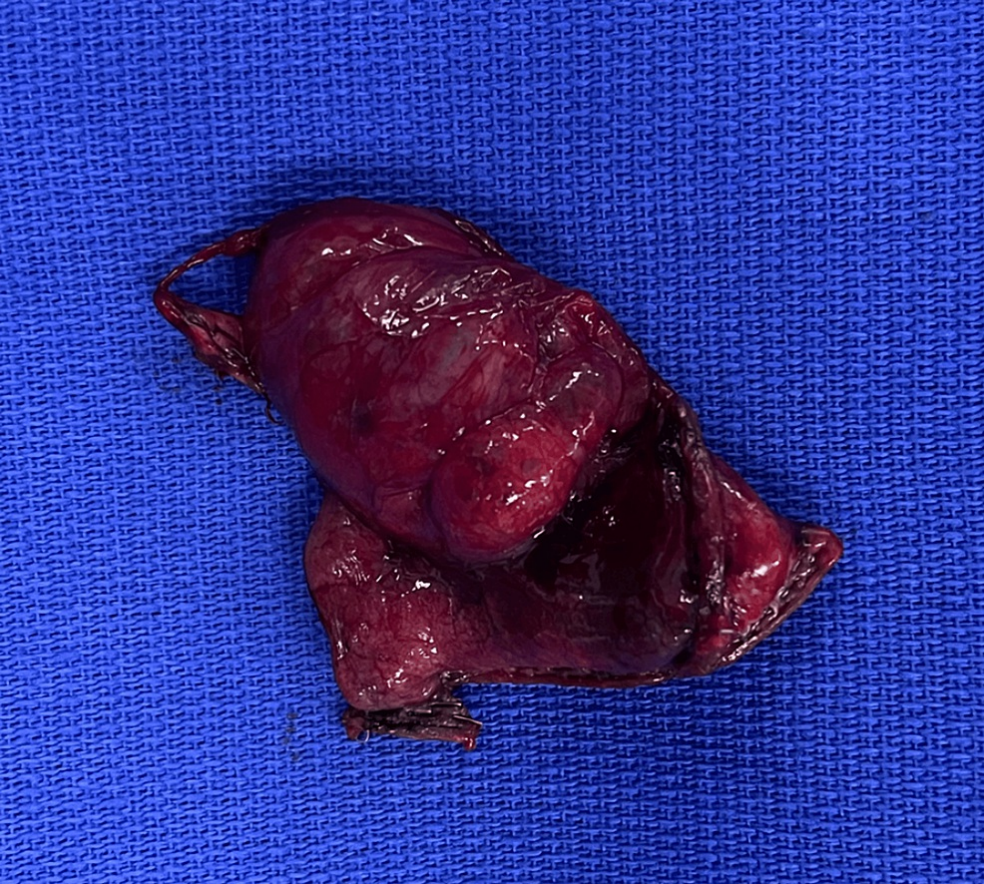
Bulla resection with view of macroscopic pathological specimen

We present a case of a 47-year-old male without a relevant history or past respiratory diseases who presented with an acute, non-complicated COVID-19 infection diagnosed by a PCR test. He started with mMRC-2 dyspnea with a non-expectant cough for four months, which was treated with 600 mg of acetylcysteine daily. His physical exploration was normal at the external consult after the medical management. We then proceeded to the diagnostic approach with a thoracic CT scan showing a dilatation of the aerial homogenous space and a well-defined anterior left pericardiac level of 4.6 cm × 4.4 cm × 3.5 cm dimensions of thin wall, with a 1 mm thickness (Figure 1). A left pericardiac bulla was diagnosed, with no abnormalities detected in the right lung. Resection in the wedge of the left lung with video-assisted thoracoscopy surgery (VATS) was performed under the preoperative diagnosis of pulmonary symptomatic bulla found in the fifth lung section (Figures 2-3). The macroscopic examination of the lung segmentectomy resection, performed using an automatic surgical stapler, revealed dimensions of 6 cm × 5 cm × 6 cm (Figure 4).

**Figure 5:**
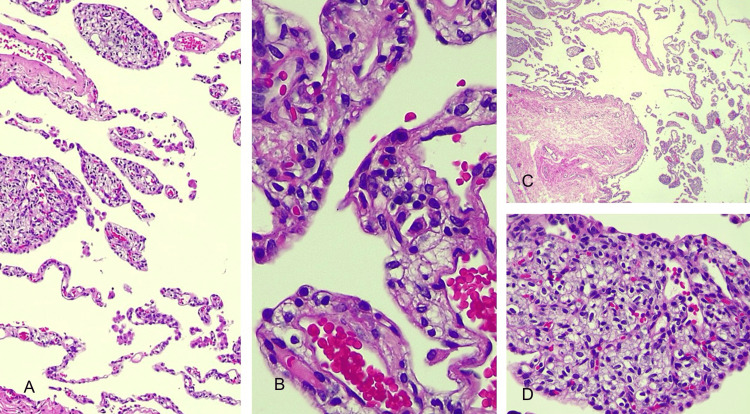
(A-D) Histopathological findings showing alveolar septa (A) with a fragment of emphysematous bulla wall (C) and intraluminal presence of vascular/capillary structures with a papillary appearance and with proliferation of associated pneumocytes reminiscent of placental chorionic villi (B/D)

The pathological exam of the solid sites by optic microscopy indicated general emphysematous changes with alveolar wall destruction, besides numerous tissues substituted by structures similar to chorionic villi. The villi structure was found adjacent to or, at times, adhered to the destroyed alveolar wall and the interlobular partitions. There were not any positive cells for human chorionic gonadotropin. The chest tube was removed five days post-surgery, and the patient was free of any surgical complications. The anatomic-pathological examination of the surgical piece reported papillary proliferation outbreaks of pneumocytes with histological details of pulmonary placental transmogrification (Figure 5).

## Discussion

It is a rare benign cystic lesion, first described by McChesney in 1979 [1]. This disease represents a challenge for both clinical and pathological diagnosis [5]. Its name comes from the similarity between chorionic villi and the lesion’s topography because, seen microscopically, it resembles the placenta with cystic spaces filled with papillary structures but without the biological properties of the placenta [6-7]. Notably, when testing pneumocytes from our anatomical piece resection, we found negative results for human chorionic gonadotropin. Pathologically, the disease shows papillary structures similar to placental villi surrounding the pulmonary epithelium [8]. Optic microscopy of our lung lesion showed general emphysematous changes with alveolar wall destruction and numerous tissue was changed structures similar to chorionic villi. It is considered a histological variant of bullous emphysema, where the alveoli resemble the chorionic villi of the placenta, and is a characteristic finding [1-8].

Its clinical presentation varies from asymptomatic to respiratory insufficiency, depending on an existing comorbidity or underlying disease [8]. Even though our patient has no known comorbidities, as we mentioned before, we debuted with mMRC-2 dyspnea, accompanied by a non-expectant cough for four months. Also, our patient had no emphysematous disease because this was not a frequent presentation of the disease.

Imaging studies of the lung, such as plain chest radiographs or CT scans, mainly show bullous changes and rarely present as cysts or nodules [8]. They are often described as incidental tumors; however, the most frequent finding is unilateral bullous emphysema, or lipomatosis, commonly associated with pulmonary fibrochondromatous hamartomas [8]. In our patient, the presentation of the bulla was unilateral in the pericardial lung segment. Radiologically, differential diagnoses of the lesions include cystic, bullous emphysema, especially giant bullous emphysema, and solitary pulmonary lung nodules [8-9].

Earlier classifications grouped radiological findings into three categories. The first and most common is the bullous emphysema pattern, followed by a mixed pattern characterized by a cystic lesion of the thin wall with a nodule, and the third and less common is a solitary nodule pattern, which in rare cases would contain air and fat [8-10].

## Conclusions

Pulmonary placental transmogrification (PPT) is a rare disease, and the preoperative diagnosis is a challenge. This case highlights the importance of keeping this entity in the differential diagnosis in patients with a previous COVID-19 infection and chronic cough. The PPT must be managed by a multidisciplinary team with the definitive treatment of surgical resection. The surgical management must be preferably done with minimal invasion, either with video-assisted thoracoscopic surgery (VATS) or robotic-assisted thoracoscopic surgery (RATS), especially because of the excellent prognosis and curability of surgical intervention.

## References

[1] Kim JW, Park IH, Kwon W, Eom MS, Kim YJ, Oh JH (2013). Placental transmogrification of the lung. Korean J Radiol.

[2] Ortiz S, Tortosa F (2017). Pulmonary placental transmogrification: the last 16 years in a reference centre. Rev Port Pneumol (2006).

[3] Horsley WS, Gal AA, Mansour KA (1997). Unilateral giant bullous emphysema with placental transmogrification of the lung. Ann Thorac Surg.

[4] Vila L, Reginatto A, Rivero H, Rayá M, Guma G, Patané AK (2020). [Placental transmogrification of the lung. Atypical presentation of the bullous emphysema]. Medicina (B Aires).

[5] Foschini G, Rodríguez CM, Rubio MM, Baldo X (2022). Placental transmogrification of the lung. Arch Bronconeumol.

[6] Jenkins JM, Attia RQ, Green A, Cane P, Pilling J (2016). A case of pulmonary placental transmogrification. Asian Cardiovasc Thorac Ann.

[7] Shapiro M, Vidal C, Lipskar AM, Gil J, Litle VR (2009). Placental transmogrification of the lung presenting as emphysema and a lung mass. Ann Thorac Surg.

[8] Ma DJ, Liu HS, Li SQ, Zhou XY, Cui YS, Wu HW, Zhou WX (2017). Placental transmogrification of the lung: case report and systematic review of the literature. Medicine (Baltimore).

[9] Dunning K, Chen S, Aksade A, Boonswang A, Dorman S (2008). Placental transmogrification of the lung presenting as tension pneumothorax: case report with review of literature. J Thorac Cardiovasc Surg.

[10] Ferretti GR, Kocier M, Moro-Sibilot D, Brichon PY, Lantuejoul S (2004). Placental transmogrification of the lung: CT-pathologic correlation of a rare pulmonary nodule. AJR Am J Roentgenol.

